# Supplementary material for: AQP3 and AQP9—Contrary Players in Sepsis?
Source: Int J Mol Sci. 2024 Jan 19;25(2):1209. doi: 10.3390/ijms25021209 (PMC10816878; doi:10.3390/ijms25021209)
Supplement: Supplementary file 1 [file ijms-25-01209-s001.zip › ijms-2794406-supplementary.pdf]

Suplementary figures

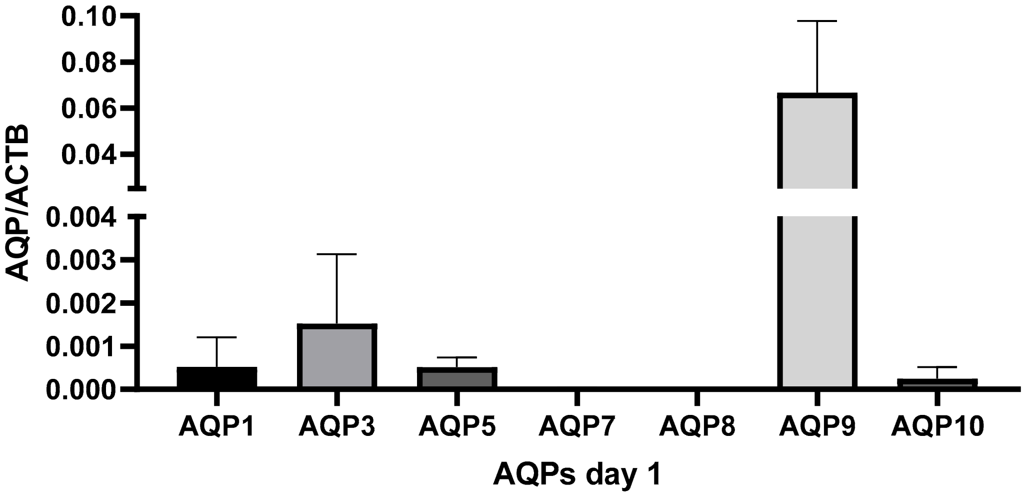

Figure S1. Aquaporin expression of different aquaporins in whole blood samples of septic patients. AQP expression was quantified relatively to ACTB (n = 85)

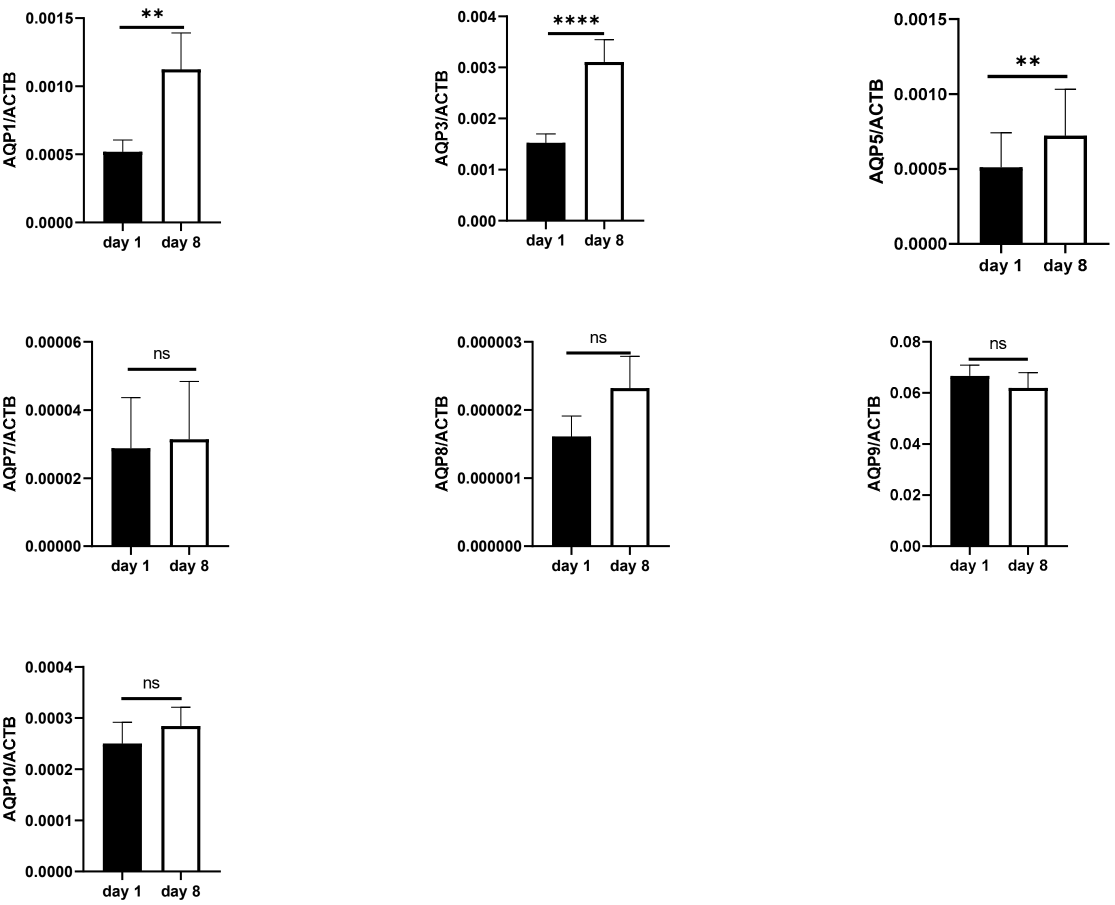

Figure S2. Aquaporin expression in whole blood samples of septic patients on day 1 and day 8 after sepsis diagnosis Aquaporin expression was analyzed in the time course of sepsis. AQP1 (** p=0.0043;) and AQP3 (**** p<0.0001) expression was more than doubled, when comparing day 8 with day 1 ADQP5 expression was also increased (** p=0,0022). A slight increase could be detected in AQP8 expression (p=n.s.), whereas AQP7, AQP9 and AQP10 expression was not altered in the time course of sepsis.
